# Therapeutic effect of a *Chlamydia pecorum* recombinant major outer membrane protein vaccine on ocular disease in koalas (*Phascolarctos cinereus*)

**DOI:** 10.1371/journal.pone.0210245

**Published:** 2019-01-07

**Authors:** Sharon Nyari, Rosemary Booth, Bonnie L. Quigley, Courtney A. Waugh, Peter Timms

**Affiliations:** 1 University of the Sunshine Coast, Sippy Downs, Queensland, Australia; 2 Australia Zoo Wildlife Hospital, Beerwah, Queensland, Australia; Midwestern University, UNITED STATES

## Abstract

*Chlamydia pecorum* is responsible for causing ocular infection and disease which can lead to blindness in koalas (*Phascolarctos cinereus*). Antibiotics are the current treatment for chlamydial infection and disease in koalas, however, they can be detrimental for the koala’s gastrointestinal tract microbiota and in severe cases, can lead to dysbiosis and death. In this study, we evaluated the therapeutic effects provided by a recombinant chlamydial major outer membrane protein (MOMP) vaccine on ocular disease in koalas. Koalas with ocular disease (unilateral or bilateral) were vaccinated and assessed for six weeks, evaluating any changes to the conjunctival tissue and discharge. Samples were collected pre- and post-vaccination to evaluate both humoral and cell-mediated immune responses. We further assessed the infecting *C*. *pecorum* genotype, host MHC class II alleles and presence of koala retrovirus type (KoRV-B). Our results clearly showed an improvement in the clinical ocular disease state of all seven koalas, post-vaccination. We observed increases in ocular mucosal IgA antibodies to whole *C*. *pecorum* elementary bodies, post-vaccination. We found that systemic cell-mediated immune responses to interferon-γ, interleukin-6 and interleukin-17A were not significantly predictive of ocular disease in koalas. Interestingly, one koala did not have as positive a clinical response (in one eye primarily) and this koala was infected with a *C*. *pecorum* genotype (E’) that was not used as part of the vaccine formula (MOMP genotypes A, F and G). The predominant MHC class II alleles identified were DAb*19, DAb*21 and DBb*05, with no two koalas identified with the same genetic sequence. Additionally, KoRV-B, which is associated with chlamydial disease outcome, was identified in two (29%) ocular diseased koalas, which still produced vaccine-induced immune responses and clinical ocular improvements post-vaccination. Our findings show promise for the use of a recombinant chlamydial MOMP vaccine for the therapeutic treatment of ocular disease in koalas.

## Introduction

*Chlamydia pecorum* is responsible for causing debilitating disease in the koala (*Phascolarctos cinereus*) infecting the urinary tract, reproductive tract, and ocular sites [1]. Infections at the urinary and reproductive sites can cause cystitis and reproductive disease, which can often lead to infertility [1–4]. Infections at the ocular site of koalas can cause kerato-conjunctivitis, which if left untreated, can lead to blindness [1, 2, 5]. Clinical signs of ocular disease in koalas can range broadly from mild to severe and can present either unilaterally or bilaterally. Typically, signs of clinical ocular disease in koalas include redness and inflammation of the conjunctiva, proliferation of the conjunctival tissue and corneal scaring [1, 2]. Inflammation and proliferation of conjunctival tissue can become quite pronounced, swelling beyond the lid margins and obscuring the eye completely [2]. Additionally, the presence of a mucoid or purulent ocular discharge can become encrusted around the eye resulting in the koala’s inability to open its eye [2]. This can all lead to rubbing and scratching of the inflamed ocular site, which can additionally put the koala at risk of sustaining further damage and injury to the eye. Other species of *Chlamydia* can also infect the eyes of their host, with the most important being *C*. *trachomatis* which infects the eyes in humans, causing debilitating disease and is recognised as the number one cause of preventable blindness [6, 7].

The mechanisms that drive the progression of a chlamydial infection to a diseased state in koalas remains unclear, with characteristics of the infecting *Chlamydia* strain, host genetic factors and host immune response being three factors implicated [8]. Koalas are known to be infected with a genetically diverse range of *C*. *pecorum* genotypes [9–11] and reports suggest that particular *C*. *pecorum* genotypes may be more prevalent at distinct anatomical sites [10, 12]. This is similar to what is reported in humans, where different *C*. *trachomatis* strains have different virulence characteristics and different tissue site trophisms [6, 13–15]. It has also been shown that koalas residing at different geographical locations are infected with different *C*. *pecorum* genotypes, with varying levels of disease observed [9, 11]. Koalas located in Victoria (southern Australia) have been identified as predominantly being infected with *C*. *pecorum* genotype B and to a lesser degree, genotypes C, F, L, M and N [10], and report a relatively low prevalence of disease [16]. In comparison, *C*. *pecorum* infected koalas located in Queensland (north eastern Australia) have been identified with genotypes A, E’, F, G and H [9] and report a higher prevalence of disease among wild koala populations [11, 12, 17]. Furthermore, it remains unclear what bearing the level of chlamydial load has on the clinical disease outcome, with reports identifying some diseased koalas with high chlamydial loads while other diseased koalas have no detectable levels of a current chlamydial infection [12].

From the host side, host genetic variability of the koala, such as differences in Major Histocompatibility Complex (MHC) gene alleles, will impact which chlamydial antigens are presented to T-cells in different koalas, influencing the immune response mounted and clinical disease outcome achieved [8, 18]. This has been demonstrated with studies identifying individual MHC class II gene alleles and linking them to antibody production, chlamydial load and disease outcome within wild koala populations [8, 18]. Humoral responses are important immune factors to consider when evaluating the reduction in inflammation and swelling of the conjunctival tissue. The impact of antibodies, in particular immunoglobulin-A (IgA) at the mucosal site, is thought to: a) inhibit bacterial adherence to epithelial cells [19], b) neutralise intracellular pathogens [20], and c) remove bacterial cells from the epithelium [21]. Importantly, secretory IgA (sIgA) plays a role in intracellular neutralisation, reduction of bacterial-induced pro-inflammatory responses, and contributes to localised homeostasis and reduced inflammation and swelling [20, 22]. Finally, the important role of IgG has been demonstrated in post-vaccinated koalas showing *Chlamydia*-specific neutralisation abilities [23] and possible protection against chlamydial infection [24].

Cell-mediated immune responses are pivotal in inflammatory responses, with interferon-gamma (IFN-γ) providing a protective role via eradication of chlamydial pathogens [25–28]. In addition to this, using the mouse model, it has been demonstrated that IFN-γ was required to prevent the progression of disease post chlamydial infection [29]. Interleukins (IL) also have effects, with the dual role played by IL-6 as both a pro and anti-inflammatory cytokine providing an additional mechanism in controlling inflammation and maintaining homeostasis [30, 31]. IL-6 has further been shown to be important for reducing and limiting a *C*. *muridarum* infection in the genital tract of mice [32]. The role of IL-17A is via a pro-inflammatory response with increased expression of IL-17A associated with active *C*. *trachomatis* [33], and higher levels of IL-17A have also been observed in diseased koalas compared to non-diseased koala [34]. In addition to this, IL-17 has been shown to play a protective role against bacterial infections [35] and could be essential for inducing immune responses against bacterial infections [36]. Maher *et al*. (2016), have further noted that IL-17A expression was significantly increased in koala retrovirus-B (KoRV-B) positive koalas [37].

KoRV is a gamma retrovirus identified in both captive and wild koala populations [38]. The prevalence of KoRV varies, depending on geographical location, with a higher prevalence identified in Queensland koalas compared to Victorian koalas [39]. Variations of KoRV genotype are also seen within koala populations with KoRV-B being previously linked to disease outcome [40]. Increased levels of KoRV have been identified in koalas suffering from leukaemia or lymphoma [41] and KoRV-B has further been associated with chlamydial disease outcomes in wild koala populations [40, 42]. Importantly, KoRV-B has been implicated as a high-risk factor for the progression to ocular disease in *C*. *pecorum* infected koalas [8].

*C*. *pecorum* infections in koalas are a major threat to long-term viability of wild koala populations, with debilitating disease outcomes and reduced fecundity, which ultimately threatens their survival. Further investigation is still required to understand the mechanisms driving the progression of chlamydial infection to disease and monitoring clinical changes as disease progresses through the various stages. Currently, treatment of chlamydiosis in koalas is by antibiotics [43–45]. Although antibiotics have been shown to be beneficial in the treatment of *C*. *pecorum* infected koalas [43], evidence has emerged that antibiotic treatment alters the microflora of the koala’s gastrointestinal tract, which is essential for the digestion of eucalyptus leaves [46, 47]. Consequently, the use of antibiotics in koalas has often been associated with gastrointestinal dysbiosis [48, 49], which can be fatal [50]. As well, antibiotic treatment has been reported to only be effective in koalas with low levels of chlamydial infections [43]. Alternatives to antibiotic treatment of chlamydial infections in koalas is currently not available despite being desperately needed; a chlamydial vaccine is a logical choice to treat disease and overcome unwanted side effects.

Our group has progressively been assessing the immunological responses to a recombinant major outer membrane protein (rMOMP) vaccine in koalas, with promising results [23, 24, 51–58]. In short, this work has demonstrated, a) *Chlamydia*-specific vaccine induces both systemic IgG and mucosal IgA antibodies [23, 24, 51, 53–56, 59], b) strong cell-mediated responses, including the production of IFN-γ and IL-17A [23, 54, 58], and c) immune cross recognition of other koala-*C*. *pecorum* genotypes [51–53, 59]. Additionally, a pilot study revealed a potential therapeutic effect of a rMOMP vaccine for ocular diseased koalas [57]. The current project aimed to significantly extend previous preliminary work by evaluating both humoral and cell-mediated responses and the possible influences of both bacterial and host genetics on ocular diseased koalas. This trial showed that it is possible to vaccinate diseased koalas and reverse their clinical disease at the ocular site. Immune parameter analysis suggests that one key positive vaccine response involved was a strong ocular IgA response.

## Materials and methods

### Animals

**Fig 1:**
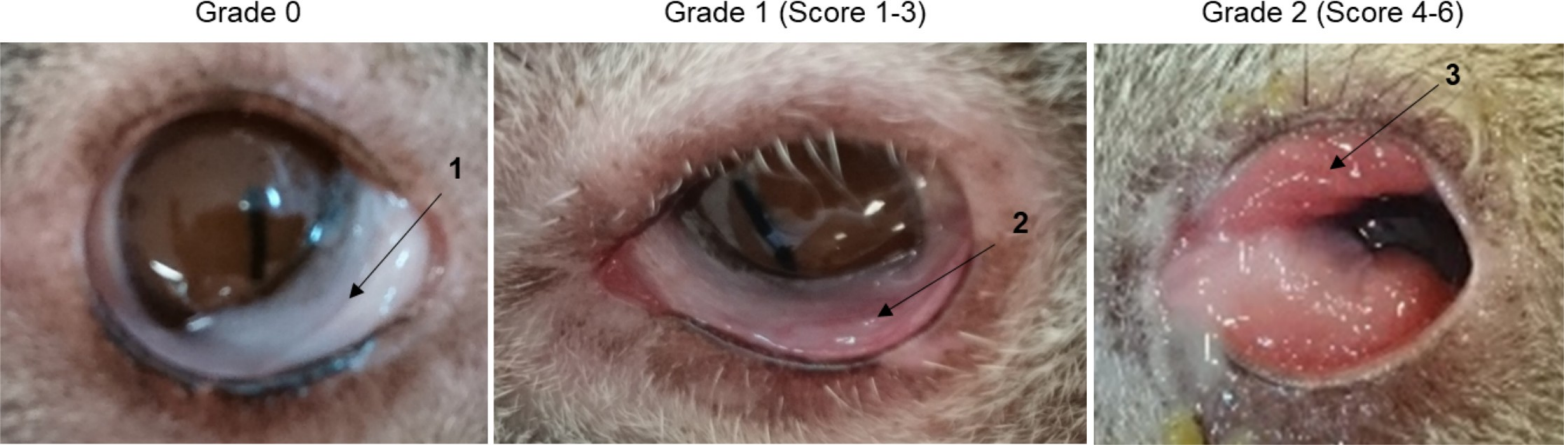
Range of clinical ocular disease observed in koalas infected with *Chlamydia pecorum*. (1) Grade 0, clinically healthy conjunctiva from koalas with no evidence of ocular disease. (2) Grade 1 ocular disease with mild redness and swelling of the conjunctiva. (3) Grade 2 ocular disease with redness, swelling and proliferation of the conjunctiva.

Seven koalas (3 males, 4 females) with either unilateral or bilateral ocular disease were selected from wild populations residing in south east Queensland, Australia. They were recruited into the trial after presentation to Australia Zoo Wildlife Hospital (AZWH) requiring treatment for chlamydial ocular disease. All koalas received a full veterinary examination upon presentation and only koalas with either grade 1 or grade 2 ocular disease (Fig 1), as assessed by the veterinarian, were recruited into the trial. All koalas had no signs of urogenital tract disease, neither overtly nor sub-clinically and were housed at AZWH for the seven-week duration of the trial. Koalas were housed individually in enclosures that comply with the Code of Practice for Wildlife Care (Queensland) and either met or exceeded zoo standards for koala enclosures. All koalas were individually checked daily by an experienced wildlife veterinary nurse. At the completion of the trial, if required, koalas received further treatment, and when possible were released back to the wild where they were originally located. All procedures relating to this study were approved by the University of the Sunshine Coast (USC) Animal Ethics Committee (Animal Ethics permit number AN/S/15/40) and by the Queensland Government (Scientific Purposes Permit number WISP16501015).

### Veterinary examination and sample collection

Koalas underwent a comprehensive veterinary examination under anaesthesia on initial presentation to AZWH, and then weekly for six weeks. During each of these examinations, the ocular site of each eye was examined and assessed by the veterinarian who graded the severity and any changes to the ocular disease state. Since ocular disease grading categories can be phenotypically broad, to further categorise changes in clinical state during this trial, we subdivided the grading categories. Healthy eyes were assigned to grade 0. Eyes with redness and swelling of the conjunctiva, with or without mucoidal discharge, were assigned to grade 1 with a score of 1–3, depending on severity. Eyes that presented with proliferation of the conjunctiva (lumps appearing within the conjunctival tissue), along with increased redness and swelling, and with or without purulent discharge, were assigned to grade 2 with a score of 4–6, depending on severity (Fig 1). Changes in ocular disease state were recorded independently for each eye. Biological samples were collected at each examination, pre-vaccination and weekly for six weeks. Whole blood (5 mL) was collected and placed into EDTA collection tubes (Interpath Services) and stored at 4°C until centrifugation where the plasma was then removed and stored at -20°C. The remaining blood was processed immediately for the collection of peripheral blood mononuclear cells (PBMC) (detailed below). Two swab samples were collected from each eye containing cells from the epithelial surface (Aluminium rayon swabs; Copan). One ocular swab was placed into 1 mL PBS containing 1% protease inhibitor cocktail (PIC) and stored at -20°C for later use in the IgA ELISA assay (detailed below). The other ocular swab was placed back into the sterile collection tube and stored at -20°C for later use in the quantification of chlamydial load by PCR (detailed below).

### Vaccine preparation and administration

The vaccine antigen consisted of three different genotypes of *Chlamydia pecorum* recombinant major outer membrane proteins (rMOMP) A, F and G [9]. The method used for purifying the rMOMP proteins is as previously described by Kollipara *et al*. (2012) [51]. The vaccine antigen containing rMOMPs A, F and G (50 μg each rMOMP protein) was combined with Tri-Adjuvant, containing Poly I;C (250 μg), Host Defence Peptide-Innate Defence Regulator IDR-1002 (500 μg), and Polyphosphazene EP3 (250 μg) (VIDO-Intervac, University of Saskatchewan, Canada), to a total vaccine volume of 500 μL with sterile endotoxin-free PBS. Each vaccine was prepared in a sterile endotoxin-free amber glass vial, stored on ice and administered within 2 hours of preparation. Post veterinary examination and sample collection, koalas received a single subcutaneous injection. No antibiotic treatment was administered to any of the koalas during the trial period.

### Koala specific *Chlamydia pecorum* IgG ELISA

*C*. *pecorum*-specific systemic IgG titres were determined from koala plasma by ELISA assay targeting using either recombinant MOMP G protein or heat inactivated semi-purified *C*. *pecorum* G elementary bodies (EBs) (purified as per [60]). Initially, 96 well plates (Greiner Bio-One medium binding) where coated with 50 μL of carbonate-bicarbonate coating buffer containing either 2 μg/well of recombinant MOMP G or 50,000 IFU/well of heat inactivated semi-purified *C*. *pecorum* G EBs and incubated at 4°C overnight. Wells were emptied and then coated with 100 μL per well of blocking buffer consisting of 5% skim milk in PBS containing 0.01% Tween-20 and incubated for an additional 2 hours at 37°C. Post incubation, wells were emptied and 1:3 serially diluted plasma, with dilutions starting at 1:50, was added in duplicate and incubated for 1 hour at 37ºC. Next, wells were washed 3 times with PBS containing 0.05% Tween-20 and coated with 50 μL/well of sheep anti-koala IgG diluted 1:8,000 in PBS containing 0.01% Tween-20, followed by incubation for 1 hour at 37˚C. Wells were again washed 3 times with PBS containing 0.05% Tween-20 and coated with 50 μL/well of HRP-conjugated donkey anti-sheep IgG diluted 1:20,000 (Abcam) in PBS containing 0.01% Tween-20, followed by incubation for 1 hour at 37°C. Wells were then washed 3 times with PBS alone then 50 μL/well of 3,3’,5,5’-Tetramethylbenzidine substrate (Sigma-Aldrich) was added and incubated at room temperature for 30 mins before stopping the reaction with 50 μL/well of 1 M sulphuric acid. Plates were read using an EnSpire Multimode plate reader (PerkinElmer) and the end point titre (EPT) was calculated as per [58].

### Koala specific *Chlamydia pecorum* IgA ELISA

The mucosal IgA response was determined from ocular swab samples stored in 1% PIC by ELISA assay targeting either using recombinant MOMP G protein or heat inactivated semi-purified *C*. *pecorum* G EBs. Initially, 96 well plates were coated with antigen and blocked as above for systemic IgG. Prepared plates received 50 μL/well of swab sample solution was added in duplicate (defrosted at room temperature and vortexed for 3 minutes prior to addition) and incubated for 1 hour at 37°C. Post incubation, wells were washed 3 times with PBS containing 0.05% Tween-20 then coated with 50 μL/well of rabbit anti-koala IgA diluted 1:3,000 in PBS containing 0.01% Tween-20 and incubated for 1 hour at 37°C. Wells were then washed 3 times with PBS containing 0.05% Tween-20 and coated with 50 μL/well of HRP-conjugated goat anti-rabbit IgG (ab6721; Abcam) diluted 1:20,000 in PBS containing 0.01% Tween-20 and incubated for 1 hour at 37°C. Finally, wells were washed, colour developed and read as above for systemic IgG. The optical density (OD) was measured at 450 nm and the absorbance value was calculated as the mean of duplicate samples minus the mean of the no sample control wells.

### PBMC purification and antigen stimulation

Peripheral blood mononuclear cells (PBMCs) were extracted from whole blood for further use in determining cell-mediated responses. Whole blood sample was centrifuged at 1000 rpm for 5 mins at 15°C. Plasma was removed and stored at -20°C. The remaining cellular fraction were resuspended in an equal volume of PBS. The cell suspension was then overlayed onto 10ml of Ficoll-paque (GE Healthcare, Australia) and centrifuged at 400 g for 25 min, brakes off, at 18°C. Cells were removed from the middle buffy coat fraction, placed into 10 ml of PBS, and centrifuged at 400 g for 10 min at 18°C. Pelleted cells were washed three times by resuspending in 10 mL PBS, followed by centrifugation at 400 g for 10 min at 18°C. Final cell pellets were resuspended in 1ml RPMI 1640 media (Gibco Life technologies, Australia) containing 10% heat-inactivated foetal calf serum (Life technologies, Australia), 120 μg/mL streptomycin (Sigma-Aldrich, Australia) and 50 μg/mL gentamycin (Gibco Life technologies, Australia). An aliquot of cell suspension was mixed with Trypan blue solution (Gilco Life technologies, Australia) at 1:10 dilution and placed onto a haemocytometer for counting using light microscope. The remaining PBMC cell suspension was diluted to 2 x 10^6^ cells/mL with RPMI 1640 media containing 10% heat-inactivated foetal calf serum, 120 μg/mL streptomycin, and 50 μg/mL gentamycin and transferred to a 96 well plate (Greiner Bio-One medium binding) at 100 μL/well. Half the PBMCs were used as control and the other half were stimulated with 100 μL/well of 2 μg/100 μL of rMOMP in RPMI 1640 media containing 10% heat-inactivated foetal calf serum, 120 μg/mL streptomycin and 50 μg/mL gentamycin and incubated at 37°C in 5% CO_2_ for 12 hours. Following 12 hour incubation, cells were collected and centrifuged at 2,000g for 10 minutes at 18°C. PBMCs were then suspended in 1mL of TRIzol reagent (Invitrogen, Australia) and store at -80°C. PBMCs were not extracted for koala K1.

### RNA extraction from PBMCs and reverse transcription

For the extraction of RNA from PBMCs, 200 μL of chloroform (Sigma-Aldrich, Australia) was first added to PBMCs suspended in TRIzol, then centrifuged at 12,000 g for 15 mins at 4°C. The top aqueous layer was removed and added to an equal volume of 100% ethanol. All further RNA extraction procedures were as per manufacturer’s instructions for RNeasy mini kit (Qiagen, Australia). For the elimination of DNA, DNase I (Sigma-Aldrich, Australia) was used, as per manufacturer’s instructions. Reverse transcription was performed using iScript reverse transcription supermix (Bio-Rad, Australia), as per manufacturer’s instructions. cDNA was then stored at -20°C until utilised in qRT-PCR for gene expression.

### Quantification of cytokine gene expression

cDNA, extracted from PBMCs, was used to measure the level of koala gene expression using quantitative real-time PCR (qPCR). Gene expression of IFN-γ, IL-6, IL-17A was measured and compared to the house-keeping gene GAPDH. All forward and reverse primers are listed in Table 1. Each PCR reaction contained 2 μL of cDNA template added to a mastermix containing, 1x Quantitect SYBR Green (Qiagen), 0.3 μM of each forward and reverse primer and molecular grade water making a final volume of 20 μL per reaction. For PCR amplification, there was an initial denaturation at 95°C for 15 mins, followed by 40 cycles of 94°C for 15 secs, 59°C for 30 secs and 72°C for 30 secs, followed by melt curve analysis (72°C to 95°C in 0.5°C increments). All reactions were performed in duplicate and carried out on a Rotor-Gene Q 5-plex HRM platform (Qiagen). Koala gene expressions were normalised to GAPDH using the 2^-ΔΔ*C*^_T_ equation (where *C*_*T*_ is threshold cycle): Δ*C*_T_ = (*C*_T_ of IFN-γ, IL-6 or IL-17A gene–*C*_T_ of GAPDH gene) and ΔΔ*C*_T_ = (Δ*C*_T_ of stimulated sample - Δ*C*_T_ of unstimulated sample) [61, 62].

**Table 1:** PCR primer sequences for gene expression.

Gene Target	Primer orientation	Sequence (5'-3')	Amplicon size (bp)	Reference
IFN-γ	Forward	TGAACATGATGGATCGTTGG	182	[63]
IFN-γ	Reverse	CACTTTGCTGGCAGTGTTGT		This study
IL-6	Forward	CACCTGTTTGGCTTTTAGCA	187	This study
IL-6	Reverse	GCAGAGCTTGTATGGCTCCT		This study
IL-17A	Forward	TCCCTAATGAGGATGCCAAC	173	This study
IL-17A	Reverse	TTGGAAGGAAGTGGAGCAGT		This study
GAPDH	Forward	AACTTTGGCATTGTGGAAGG	188	This study
GAPDH	Reverse	GTGAGCTTCCCATTCAGCTC		This study

Gene targets with corresponding forward and reverse primer and amplicon size (bp).

### DNA extraction and qPCR quantification of *Chlamydia pecorum* load

Swab samples collected from both the left and right eyes of koalas were thawed at room temperature and added to 1.5 mL sucrose phosphate glutamate at pH 7.4 (0.2 M sucrose, 3.8 mM potassium phosphate monobasic, 8.6 mM disodium phosphate, 4.9 mM glutamic acid). Swabs were vortexed for 3 mins before 1 mL of solution was removed and centrifuged at 18,000 rpm for 20 mins. The resulting cell pellet was re-suspended in 50 μL TE buffer and heated at 95°C for 10 mins. DNA extraction was then performed using QIAmp DNA mini kit (Qiagen), as per manufactures instructions, with the exception of the proteinase K digestion being extended to 12 hours. Extracted DNA was screened for the presence and load of *C*. *pecorum* using quantitative real-time PCR (qPCR) with the forward primer: 5’ AGTCGAACGGAATAATGGCT 3’, and the reverse primer: 5’ CCAACAAGCTGATATCCCAC 3’, which targeted a 204 bp fragment of the *C*. *pecorum* 16S rRNA gene. Each PCR reaction contained 5 μL of DNA template added to a mastermix containing, 1x Quantitect SYBR Green (Qiagen), 0.5 μM of each forward and reverse primer and molecular grade water making a final volume of 20 μL per reaction. For PCR amplification, there was an initial denaturation at 95°C for 15 mins, followed by 35 cycles of 94°C for 15 secs, 57°C for 15 secs and 72°C for 30 secs, followed by melt curve analysis (55°C to 95°C in 0.5°C increments). All reactions were performed in duplicate and samples of ˃ 50 copies/μL were considered positive. All reactions were carried out on a Rotor-Gene Q 5-plex HRM platform (Qiagen).

### *Chalmydia pecorum* ompA genotyping

Outer membrane protein A (ompA) sequencing was performed using DNA extracted from both eyes to determine the genotype infecting each ocular sites and genetic diversity among our koalas. DNA extracted from the ocular swabs was used as template for conventional PCR amplification. The forward primer: CpeNTVD3 5’-GTTCTTTCTAACGTAGC-3’, and the reverse primer: CpeNTVD4 5’-TGAAGAGAAACAATTTG-3’ were used to amplify the VD3 and VD4 regions of the ompA gene targeting the 359 bp fragment located at 670–1,028 bp region of the full length ompA gene. Each PCR reaction contained 1 μL of gDNA template added to a mastermix containing, 12.5 μL of 2x HotStar Taq (Qiagen), 0.3 mM of each forward and reverse primer and molecular grade water making a final volume of 25 μL per reaction. For PCR amplification, there was an initial denaturation at 95°C for 5 mins, then 40 cycles of denaturation at 95°C for 30 secs, primer annealing at 46°C for 30 secs, primer extension at 72°C for 30 secs, followed by a final extension at 72°C for 5 mins. All ompA sequences were determined by direct sequencing of the PCR product using CpeNTVD3/CpeNTVD4 performed by Macrogen Inc. (Korea).

### MHC class II gene identification

MHC class II DAb and DBb gene alleles were determined as previously described by Quigley *et al*. (2018) [8] with the exception that DNA was extracted from swabs collected from the eyes, as described in this paper.

### KoRV-A and KoRV-B identification

The identification of KoRV-A and KoRV-B in our trial koalas was determined as previously described by Quigley *et al*. (2018) [40] with the exception that DNA was extracted from swabs collected from the eyes, as described in this paper.

### Statistical analysis

All statistical analysis was performed using GraphPad Prism version 7 (GraphPad Software, LaJolla, CA, USA). A Wilcoxon signed rank test, and paired *t* test were used to determine the difference in clinical ocular score, mucosal IgA antibodies, and systemic IgG antibody titres between pre- and post-vaccination, as appropriate.

## Results

### Clinical improvement was observed in the eyes of all koalas at six-weeks post-vaccination

Clinical differences in the eyes of the trial koalas were examined pre-vaccination and then weekly for six-weeks to observe any pathological changes. Pathological changes were recorded as; a) changes in colour to the conjunctival tissue, b) the amount of inflammation within and around the ocular site, c) the onset or changes in the severity of proliferation seen in the conjunctival tissue, and d) the amount of discharge exuded. Where proliferation of the conjunctival tissue was present, it could be seen in either the upper or lower eyelid and predominantly towards the distal end of the conjunctiva. Mucoid and purulent discharge was seen in some koalas with purulent discharge, observed more in grade 2 ocular disease. The level of clinical disease varied between each eye and between koalas, with examples of different ocular disease stages shown in Fig 1 (Fig 1). All the koalas in this trial were from a wild population and hence there was no available history of the disease state of each koala (acute or chronic) prior to arriving at the hospital. Each eye was examined and evaluated individually, and our observations showed that post-vaccination, each eye responded independently of the other eye.

**Fig 2:**
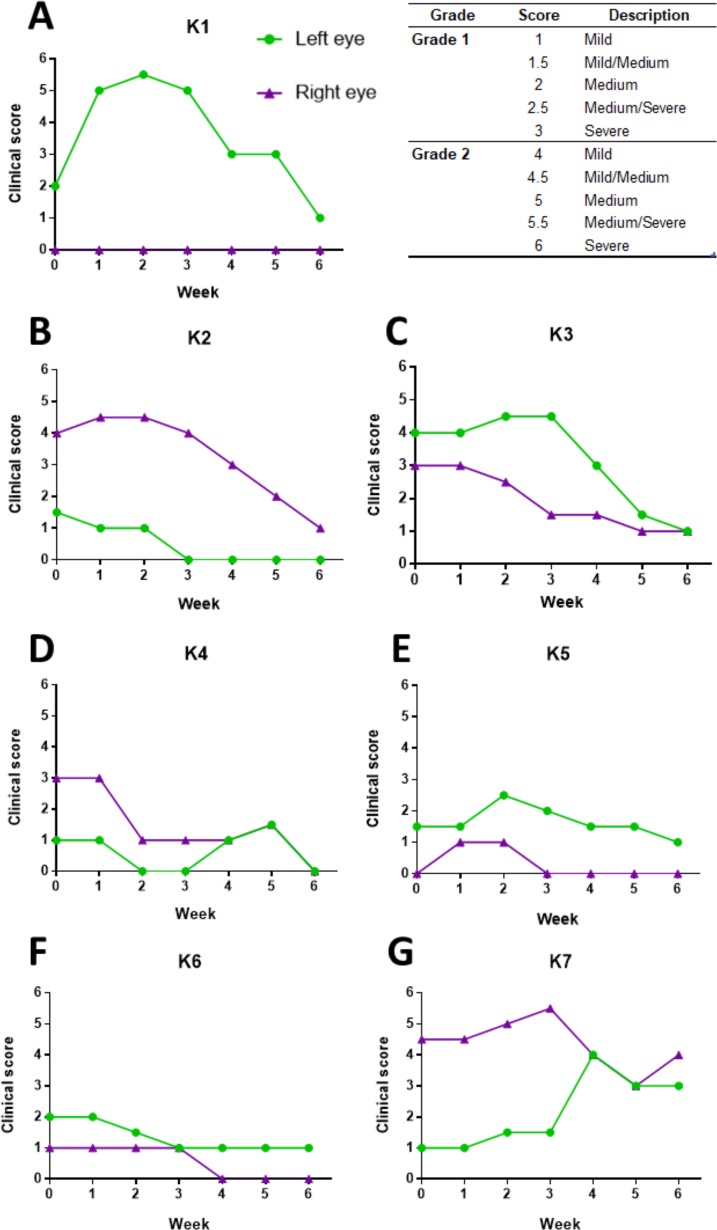
Changes in clinical ocular disease score in koalas post-vaccination. Clinical ocular disease scores measured at pre-vaccination and weekly for six weeks (1–6) post-vaccination. The clinical score represents the ocular disease state in each eye at that timepoint.

**Fig 3:**
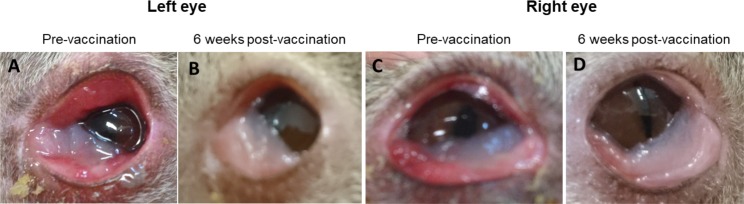
Clinical ocular disease improvement seen post-vaccination. An example of clinical ocular disease changes observed from pre-vaccination to six-weeks post-vaccination in koala K3. (A) Left eye at pre-vaccination showing inflammation, swelling and proliferation of the conjunctival tissue with a clinical score of 4. (B) Left eye six-weeks post-vaccination showing reduced inflammation and swelling, with a clinical score of 1. (C) Right eye at pre-vaccination showing inflammation and swelling of the conjunctiva with a clinical score of 3. (D) Right eye six-weeks post-vaccination showing reduced inflammation and swelling of the conjunctiva with a clinical score of 1.

All seven koalas made some level of clinical improvement, in at least one eye, after vaccination (Fig 2; Table 2). Six out of seven koalas (K1, K2, K3, K4, K5 and K6) either maintained (i.e. no worsening) or improved their ocular disease state, in both eyes, across the trial (Fig 2A, 2B, 2C, 2D, 2E and 2F). The ocular disease score average for both eyes (n = 14) at pre-vaccination, was 2.3 (SE = 0.38), and the ocular disease score average for both eyes (n = 14) at six-weeks post-vaccination, was 0.9 (SE = 0.32). Importantly, there was a highly statistically significant decrease in the average ocular disease score from pre-vaccination, to six-weeks post-vaccination (P = 0.0034). An example of the clinical ocular disease improvement post-vaccination is shown in Fig 3 (Fig 3). Variations in clinical response were observed over time and, notably, five out of seven koalas (K1, K2, K3, K5 and K7) showed an initial worsening (for the initial 1 to 2 weeks of the trial only) of diseased state, in at least one eye (Fig 2A, 2B, 2C, 2E and 2G). Despite this, from 3–4 weeks post-vaccination, we observed more general clinical improvements, which in most koalas, continued to improve out to the six-week endpoint. Five of the koalas (K1, K2, K4, K5 and K6) showed no signs of clinical disease, in at least one eye (and often both eyes) at the end of the trial (Fig 2A, 2B, 2D, 2E and 2F), while six of the koalas (K1, K2, K3, K4, K5 and K6) had a clinical ocular disease score of 1 or below in both eyes at the six-week endpoint (Fig 2A, 2B, 2C, 2D, 2E and 2F). Only one koala (K7), had a final clinical score above 1 in both eyes (Fig 2G).

**Table 2:** Koala ocular disease scores at pre-vaccination and post-vaccination.

Koala	Ocular disease score pre-vaccination	Ocular disease score six-weeks post-vaccination	Change in ocular disease score (+/-)
K1—Left eye	2	1	-1
K1—Right eye	0	0	0
K2—Left eye	1.5	0	-1.5
K2—Right eye	4	1	-3
K3—Left eye	4	1	-3
K3—Right eye	3	1	-2
K4—Left eye	3	0	-3
K4—Right eye	3	0	-3
K5—Left eye	1.5	1	-0.5
K5—Right eye	0	0	0
K6—Left eye	3	1	-2
K6—Right eye	1	0	-1
K7—Left eye	2	3	+1
K7—Right eye	4.5	4	-0.5

Change in clinical ocular disease score, as assessed by expert wildlife veterinarian, in both left and right eyes at pre-vaccination and again at six-weeks post-vaccination.

### Vaccination resulted in increased *Chlamydia pecorum*-specific systemic IgG and ocular IgA antibody levels

**Fig 4:**
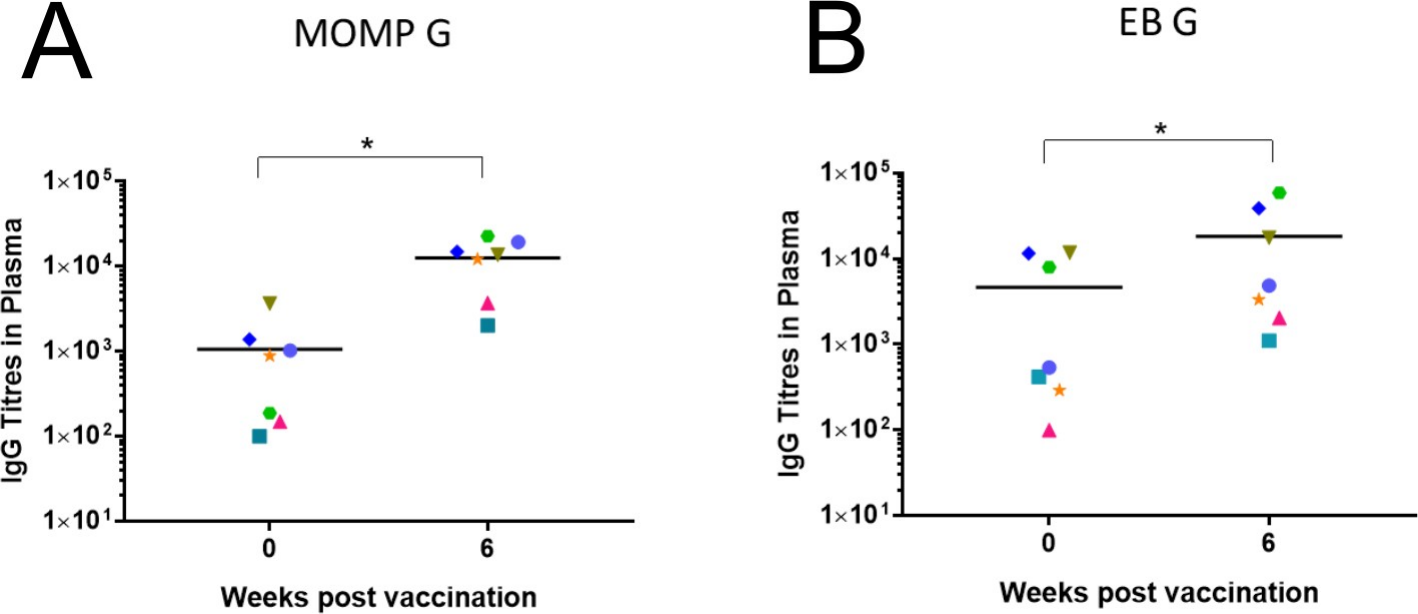
Systemic IgG antibody levels to rMOMP protein and whole *Chlamydia pecorum* elementary bodies. Systemic IgG antibodies (from plasma) were measured at pre-vaccination and six-weeks post-vaccination by ELISA assay and reported as end point titre (EPT). (A) Systemic IgG measured at pre-vaccination and six-weeks post-vaccination, against rMOMP G protein (n = 7) with a P value 0.0156. (B) Systemic IgG measured at pre-vaccination and six-weeks post-vaccination, against whole *C*. *pecorum* G EBs with a P value 0.0156.

Systemic IgG and mucosal IgA antibodies were measured against *C*. *pecorum* recombinant MOMP G protein as well as against whole *C*. *pecorum* G elementary bodies (EBs) at pre-vaccination and weekly for the duration of the trial. Although there was variation between koalas, a statistically significant increase in *C*. *pecorum*-specific systemic IgG antibodies (measured against both rMOMP G protein and whole *C*. *pecorum* EBs) was observed from pre-vaccination to six-week post-vaccination (P = 0.0156) (Fig 4; S1 Fig). Notably, koalas K1, K2 and K3 had the highest levels of systemic IgG antibodies that reacted with whole chlamydial EBs (S1A, S1B and S1C Fig), and were also among the animals with the most clinically severe natural disease.

**Fig 5:**
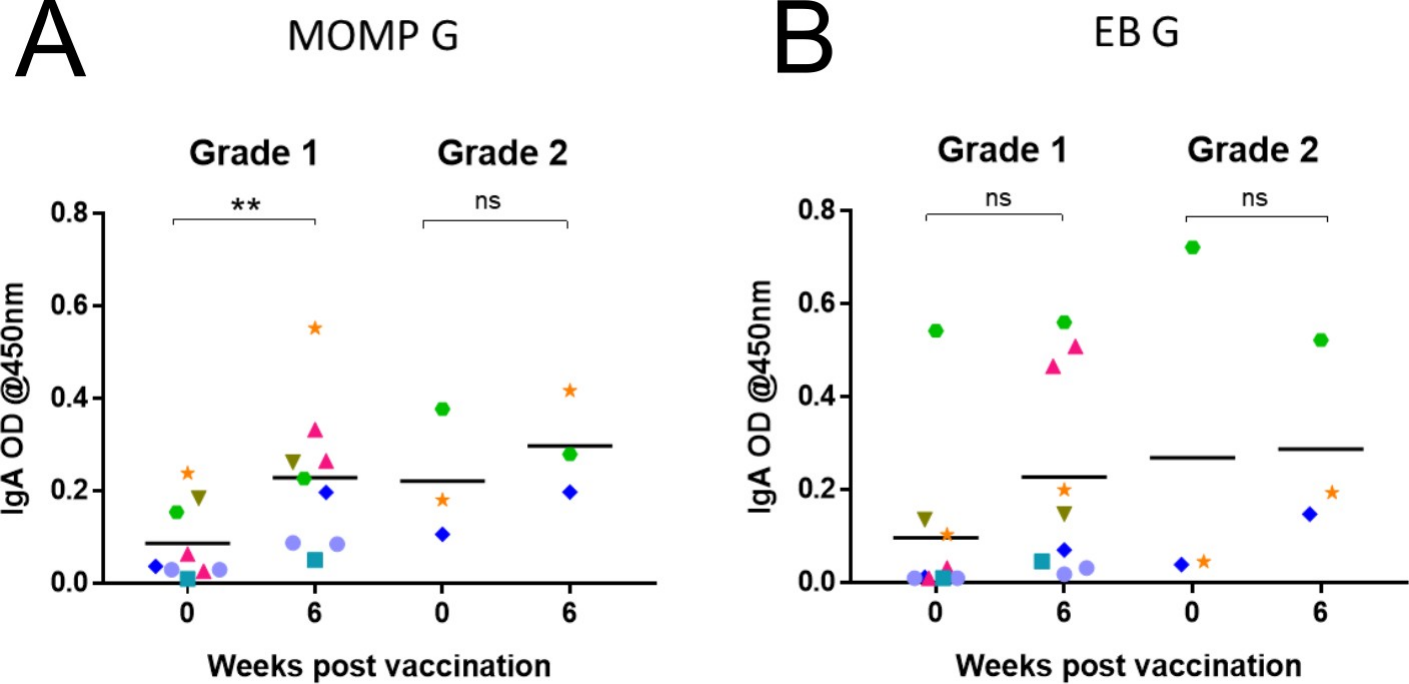
Mucosal IgA antibodies to rMOMP protein and whole *Chlamydia pecorum* elementary bodies. Mucosal *C*. *pecorum*-specific IgA antibodies (from ocular swabs) were analysed by ELISA assay and reported as optical density (OD) measured at 450 nm. IgA responses are shown against rMOMP G protein and whole *C*. *pecorum* EBs. (A) Mucosal IgA antibody responses measured at pre-vaccination and six-weeks post-vaccination, against rMOMP G in either grade 1 (P = 0.0042) or grade 2 ocular sites. (B) Mucosal IgA antibody responses measured at pre-vaccination and six-weeks post-vaccination, against EB G in either grade 1 or grade 2 ocular sites.

Mucosal IgA antibody levels were analysed in each eye, to evaluate the local vaccine-induced response. Koalas with grade 1 ocular disease at time of entry into the trial showed an increase in *C*. *pecorum*-specific mucosal IgA antibodies, in that specific eye, to both rMOMP G and *C*. *pecorum* EBs from pre-vaccination to six-weeks post-vaccination (Fig 5). This increase was statistically significant for the rMOMP G antibody response with a P value of 0.0042. (Fig 5A). The grade 2 ocular diseased sites of koalas (at pre-vaccination) also showed an increase in *C*. *pecorum*-specific mucosal IgA antibodies, in most eyes, to both rMOMP G and *C*. *pecorum* EBs, from pre-vaccination to six-weeks post-vaccination (Fig 5). Although antibody levels varied throughout the trial, all koalas (7/7) showed an increase in *C*. *pecorum*-specific mucosal IgA antibodies to both rMOMP G protein and *C*. *pecorum* EBs, in at least one eye, post-vaccination (S2 Fig). In contrast to the systemic IgG levels, most koalas (6/7; K1, K2, K4, K5, K6 and K7) had either low or no detectable levels of *C*. *pecorum*-specific IgA antibodies (measured by both *C*. *pecorum* rMOMP G protein and whole EBs) at pre-vaccination (Fig 5). The overall trend was for *C*. *pecorum*-specific IgA antibody levels to increase by week 2 and peak at around 3 to 4 weeks (S2 Fig).

### Chlamydial qPCR load was low in most koalas

All koalas were assessed for ocular *C*. *pecorum* presence and load using qPCR (S3 Fig). All koalas except one (K4 right eye) (S3D Fig) had low (< 300 copies/μL) to undetectable levels of *C*. *pecorum* pre-vaccination (S3 Fig). The *C*. *pecorum* load in an eye did not always correlate with the clinical disease observed. Some koalas with detectable *C*. *pecorum* showed no signs of ocular disease and conversely, some koalas with no detectable *C*. *pecorum* were found to have grade 1 or grade 2 ocular disease. Koala K4 had the highest initial *C*. *pecorum* load of over 30,000 copies/μL in the right eye at pre-vaccination however, this subsequently decreased to undetectable *C*. *pecorum* levels in both eyes, at six-weeks post-vaccination (S3D Fig). All other koalas (K1, K2, K3, K5, K6 and K7) recorded either no detectable levels or very low (< 600 copies/μL) detectable levels of chlamydial load at six-weeks post-vaccination for both eyes (S3A, S3B, S3C, S3E, S3F and S3G Fig).

### Cytokine expression increased post-vaccination

Cell mediated responses play an important role in the mediation of inflammatory responses. To evaluate the cell mediated responses in our vaccinated koalas, we compared the cytokine expression of IFN-γ, IL-6 and IL-17A at pre-vaccination and weekly for six-weeks post-vaccination (S1 Table). Cytokine expression was measured as fold change using RT-qPCR performed on RNA extracted from PBMCs stimulated with rMOMP G protein. There was considerable variation between koalas, expressing different levels of IFN-γ, IL-6 and IL-17A at different timepoints, post-vaccination (S1 Table). Collectively, the mean-expression, for all three cytokines (IFN-γ, IL-17A and IL-6), remained relatively similar at pre-vaccination and at six-weeks post-vaccination. For IFN-γ gene expression, we did not observe any elevated expression levels in koalas to their natural infected and diseased state, at pre-vaccination. Increases in IFN-γ expression were seen in some koalas post-vaccination, at varying time-points, but this was not consistent. Post-vaccination fold-change increases were observed to be highest in koala K4 at weeks 3 and 4 (43.5 and 25.7 respectively) (S2 Table). Similarly, we did not observe any elevated IL-6 expression levels in any koalas at pre-vaccination (S1 Table). The expression levels of IL-6 did not appear to vary in most koalas post-vaccination (S1 Table). Koala K4 again showed the highest fold-change increases post-vaccination seen from week 1 (161.0), which peaked at week 3 post-vaccination (403.0) (S2 Table). For IL-17A expression, only one koala (K7) showed a higher expression, compared to the other koalas, at pre-vaccination (S1 Table). The IL-17A expression levels observed in koala K7 then reduced from one-week post-vaccination (S1 Table). Fold-change increases were then observed in some koalas at varying timepoints post-vaccination, and once again koala K4 showed the highest fold-change increase (143.8) at two-weeks post-vaccination (S2 Table).

### Effect of chlamydial genotype of the infecting strain on response to vaccination, MHC class II allele diversity and KoRV-A and KoRV-B status

For our current study, we used ompA sequencing to type the strains of *C*. *pecorum* present in the animals in our trial, including to determine if the same strain was infecting both eyes of each koala. We also wanted to determine how well the types of MOMP protein used in the vaccine matched the infecting strains in the vaccinated animals. In our seven vaccine trial koalas, there were three different *C*. *pecorum* strains infecting the animals’ eyes (G–three koalas, F–two koalas, E’–one koala, and mixed infection–one koala) (Table 3). However, each individual koala was found to have the same *C*. *pecorum* genotype present in both eyes. Three MOMP genotypes were used in the vaccine formulation (A, F and G) and hence, six out of the seven trial koalas (K1, K2, K3, K4, K5 and K6) were infected with a *C*. *pecorum* genotype that matched the MOMPs used in the vaccine formula (Table 3). These six koalas were the same koalas that we observed clinical ocular disease improvements in by the trial endpoint. However, the koala infected with *C*. *pecorum* genotype E’ (K7), which was not used in the vaccine formula (Table 3), showed worsening clinical disease by the trial endpoint.

**Table 3:** Genetic diversity of the chlamydial strains, host MHC type and KoRV status of trial koalas.

Koala	ompA genotype	Strain included in vaccine	MHC class II DAb gene allele	MHC class II DBb gene allele	KoRV-B	Koala Age and (sex)
K1	G	Y	10, 19, 30	2, 3, 5	+	10 (M)
K2	G	Y	10, 15, 19, 21	1, 5	-	4 (F)
K3	F	Y	10, 15, 19, 21	2, 4, 5	-	3 (M)
K4	F	Y	15, 19, 21	2, 3, 5	+	1 (F)
K5	G	Y	15, 21	2, 5	-	3 (M)
K6	MIXED	Y	10, 19, 21	3	-	7 (F)
K7	E'	N	15, 19, 21	3	-	8 (F)

*Chlamydia pecorum* genotype infecting each koala, at the ocular site, as determined by ompA sequencing. Association between infecting genotype and vaccine antigen, yes (Y) or no (N). MHC class II DAb and DBb gene alleles detected in each koala showing their genetic diversity. Presence (+) or absence (-) of KoRV-B and. Koala age and sex male (M) and female (F).

To evaluate the host genetic diversity levels of our ocular diseased koalas, we determined the MHC class II DAb and DBb gene alleles [8]. We detected five different DAb alleles and five different DBb alleles amongst our seven koalas (Table 3). The most common DAb alleles detected were Dab*19 and *21, and the most common DBb allele detected was DBb*05 (Table 3). Dab*30 and DBb*01 and *04 were each only detected in one koala (K1, K2 and K3, respectively) (Table 3). All seven koalas were genetically different with no two koalas identified with the same genetic sequence.

KoRV-A and KoRV-B status was determined, at the ocular site, with particular interest in KoRV-B prevalence due to the previous association with chlamydial disease outcome [40, 42]. KoRV-A was present in all seven koalas (K1, K2, K3, K4, K5, K6 and K7) however, KoRV-B was only present in two (29%) of the koalas (K1 and K4). The two KoRV-B positive koalas were both identified as having the same DBb gene alleles (2, 3 and 5) but had different DAb gene alleles.

## Discussion

Vaccine development usually focuses on prophylactic vaccines, with relatively little effort directed towards the development of therapeutic vaccines for infectious diseases [64]. The prophylactic properties of an anti-chlamydial vaccine are no doubt key for providing long-term protection against *C*. *pecorum* infections in wild koalas. However, in addition, therapeutic effects of a vaccine could be beneficial in koalas that have progressed to disease. As a significant percentage of wild koalas have sub-clinical disease [12], the possibility of a vaccine being effective against mild disease is a promising prospect as they often go undetected and progress to severe disease. Furthermore, due to unwanted side effects following antibiotic treatment, a therapeutic vaccine would be beneficial in the treatment of koalas [47–50]. The potential to halt or even reverse clinical disease in the koala through vaccination has the potential to overcome the unwanted side effects of antibiotic usage.

Previous prophylactic studies in both captive and wild koala populations have shown that a rMOMP vaccine induces both humoral and cell-mediated immunity with long lasting (12 and six months respectively) responses [23, 24, 51, 52, 54, 55, 58, 60]. This current study expands on this research, by now evaluating the therapeutic effects of a rMOMP vaccine on ocular diseased koalas. Our current findings demonstrate that a therapeutic effect is possible, with all vaccinated koalas showing clinical ocular improvements without administering antibiotics. Additionally, we found that minor changes in the *C*. *pecorum* strain may have an impact on vaccine success, with the one koala infected with a strain not used in the vaccine formulation, exhibiting the poorest clinical response.

Pathogenesis at the ocular site of our diseased koalas varied and, notably, each eye responded independently of the other. A time lag was evident with some koalas showed an initial worsening of clinical signs within the first three weeks however, in most koalas, the clinical diseased state subsequently improved to either very low or no detectable disease by the trial endpoint. As these are koalas from a wild population, we have no known history of their previous chlamydial infections and whether the koala was in a natural progressive stage of the disease. If some koalas were in a progressive stage of disease, while others were experiencing a more stable stage of disease, then this could explain the initial increase in clinical signs seen in some koalas but not in others. This would then imply that if the clinical state was in a natural progressive stage, the vaccine has potentially halted and then reversed this natural disease progression. Furthermore, changes in the ocular disease state were found to be unrelated to the koalas’ *C*. *pecorum* load for that particular eye. This would imply that the changes in clinical ocular disease state are primarily driven by immune responses and not chlamydial load.

Immune responses should not only be specific to the prevention and clearance of the bacteria but should also contribute to the reduction in the clinical diseased state. As *C*. *pecorum* is an intracellular pathogen, infecting the epithelial cells of the mucosal surface, a primary area of interest is the mucosal immune response, including the ability to induce secretory IgA antibodies. The results from this trial show that naturally infected and diseased koalas do not produce a strong mucosal anti-chlamydial IgA response. However, following vaccination of these koalas, we observed an increase in their secretory IgA antibodies, which peaked in most koalas, at around 3–4 weeks post-vaccination. Interestingly, this was also around the time that we observed improvement of the clinical ocular diseased state of most koalas. We have previously demonstrated mucosal IgA increases in the koala at 12 and 26 weeks post-vaccination [58, 59] however, this is the first trial to analyse the mucosal IgA response in koalas at weekly intervals post-vaccination. The variation in mucosal IgA antibody response could potentially be explained by the low levels of *C*. *pecorum* load detected in our koalas. This has previously been demonstrated in a study conducted on guinea pigs where they demonstrated that the IgA response post-inoculation, was dose dependent [65]. Furthermore, we noticed that for most koalas, the eye that showed the higher IgA increase also corresponded with the eye that had the worst disease state. Regardless of grade 1 or grade 2 ocular disease, koalas made similar increases in mucosal IgA antibody response post-vaccination.

It has been shown in *C*. *trachomatis* infected humans, that there is an inverse relationship between IgA levels and chlamydial load [66], suggesting that IgA plays a direct role in decreasing and eliminating chlamydial infection at the mucosal site. The koalas in our study presented with either low or no detectable levels of *C*. *pecorum*, as determined by qPCR, with the exception of the individual K4. Low levels of *C*. *pecorum* load were not surprising, as Nyari *et al*. (2017) previously reported that the *C*. *pecorum* load was significantly lower in wild koalas with clinical disease, compared to koalas with no clinical disease [12]. Similarly, Patterson *et al*. (2015) also reported the absence of a chlamydial infection in diseased koalas from a Victorian population [16]. Notably, in our current study, koala K4 had a *C*. *pecorum* load of >30,000 copies/μL with minimal detectable levels of mucosal IgA pre-vaccination. Koala K4 subsequently increased mucosal IgA antibodies and decreased the *C*. *pecorum* load to no detectable levels post-vaccination. Taken together, this is supportive of an inverse relationship between IgA levels and chlamydial load. Similarly, in post-vaccinated challenged mice, increased levels of IgA antibodies have additionally been shown to enhance protection against a chlamydial infection [67].

Developing strong cell-mediated responses is another important factor in vaccine development [68] including anti-chlamydial vaccines [69]. In this study, we evaluated the ability of a rMOMP vaccine to induce the expression of IFN-γ, IL-6 and IL-17A. We observed that for this group of ocular diseased koalas the mean cytokine expression for all three cytokines (IFN-γ, IL-6 and IL-17A) was similar at pre-vaccination to six-weeks post-vaccination. Although we observed higher cytokine expressions in some koalas at varying time-points, post-vaccination, this was not consistent. For IFN-γ gene expression, we did not observe any elevated expression levels in koalas to their natural infected and diseased state, at pre-vaccination. This was surprising as the importance of IFN-γ in mediating immune responses for the control of chlamydial infections has been previously shown [26, 27, 70–73] with a strong correlation between the production of IFN-γ and protection against *C*. *trachomatis* [74]. In addition, IFN-γ responses have been demonstrated to be involved in both the clearance of a *Chlamydia* infection and the resistance to reinfection [75]. Importantly, the anti-inflammatory properties of IFN-γ have also been described, through the ability to modulate other pro-inflammatory cytokine production, resulting in self-regulation of inflammation [76, 77]. Given the relationship between IFN-γ expression and chlamydial infection, the low levels of IFN-γ expression seen in our vaccinated koalas could possibly be explained by the low to no detectable levels of *C*. *pecorum*. Therefore, from this data, it appears that IFN-γ is not a direct marker for infection or disease and does not seem to be responsible for the resolution of infection, in these ocular diseased koalas.

Interleukin-6 is well known as a pro-inflammatory cytokine, playing an important role in acute inflammatory responses triggered by infection and tissue damage [78–80]. The beneficial role of IL-6 has been demonstrated in a previous study where higher levels of bacterial burden along with an increased mortality rate, were observed in IL-6 depleted mice [81]. Conversely, IL-6 also has anti-inflammatory activities, which play a role in returning the host to a homeostatic state [82–84] and contributes to the processes involved in tissue repair [30]. The anti-inflammatory properties of IL-6 have been shown by Xing *et al*. (1997) where they demonstrated, in mice, how IL-6 controlled the levels of other pro-inflammatory cytokines but had no effect on anti-inflammatory cytokine responses [31]. These attributes are paramount for an anti-chlamydial vaccine to provide a therapeutic effect and, until now, have not been observed within diseased koalas. In this study, we did not observe any significant increased IL-6 expression in any koala to natural infection, at pre-vaccination. However, post-vaccination, one koala (K4) showed a high fold-change increase from one-week post-vaccination. Notably, the level of cytokine expression measured in koala K4 was not necessarily higher than other ocular disease koalas, but this koala had the lowest IL-6 expression pre-vaccination. Interestingly, this koala (K4) also showed a high *C*. *pecorum* load (> 30 000 copies/μL) at pre-vaccination and was the only koala to consistently show increased cytokine expression fold-increases, post-vaccination, to all three cytokines.

The cytokine IL-17 has been shown to be important in the defence against bacterial pathogens [36, 85]. In particular, this defensive role to intracellular bacteria was demonstrated previously by showing that IL-17 neutralised mice had significantly higher levels of chlamydial infection and severe disease outcomes [86]. Interestingly, our current study found that the one koala (K4) who had a high *C*. *pecorum* load, pre-vaccination, also expressed the lowest IL-17A response, at pre-vaccination. The IL-17A expression for koala K4 subsequently increased post-vaccination, as we observed a decrease in the *C*. *pecorum* load, along with improved clinical ocular disease state in both eyes. Evaluating the expression levels of IL-17A in the other ocular diseased koalas revealed that only one koala (K7) had elevated IL-17A expression, compared to the other koalas, at pre-vaccination. As IL-17A is a pro-inflammatory cytokine, it is not surprising that koala K7 also had the highest clinical disease score at pre-vaccination, but this does not explain the lower levels expressed in other koalas with clinical disease. Surprisingly, for koala K7 the expression levels of IL-17A decreased, post-vaccination, as clinical signs increased. All other koalas that showed increases in IL-17A expression post-vaccination, showed decreases in clinical inflammation. This is interesting and possibly indicates that IL-17A expression in koalas, may not be the main contributor to inflammation at the conjunctival site, despite reports of active trachoma in children being associated with increased expression of IL-17A at the conjunctival site [33]. This study observed cytokine expression in ocular diseased koalas and then again post-vaccination however, due to the small group of koalas used in this study, it is not possible to draw any conclusions. Additionally, cell-mediated responses were measured as systemic responses and a different profile of cytokine expression could therefore be shown at the mucosal site and should be assessed in the future.

Another interesting finding in this study was when we determined the strain of *C*. *pecorum* at the ocular site, as determined by ompA sequencing. We found that the one koala (K7) who showed the least amount of clinical improvement was infected with genotype E’. Genotype E’ was not used as part of the vaccine formulation. This was interesting, as we have previously shown that our rMOMP vaccine can induce antibodies in post-vaccinated koalas that cross-recognise other *C*. *pecorum* genotypes [59] however, this has not been shown for genotype E’. Additionally, we have previously reported, in an epidemiology study conducted on koalas in south east Queensland, that koalas infected with genotype E’ were more likely to have disease than koalas infected with genotype G [12]. Surprisingly, genotype E’ has 99.7% similarity to genotype F, which was used in the vaccine formulation. There are four single nucleotide differences between genotypes E’ and F, two synonymous and two non-synonymous changes. The relevance of minor ompA gene sequence changes and any subsequent impact on pathogenicity has been outlined previously by Sturdevant *et al*. (2010) [87] who identified single frameshift mutations within *C*. *trachomatis* serovar D. They showed that these single mutations within *C*. *trachomatis* serovar D resulted in different virulence factors which influenced the duration of the diseased state. It is therefore recommended that future *C*. *pecorum* MOMP vaccine studies consider incorporating a broader variety of MOMP genotypes focusing on specific genotypes identified within particular koala populations.

The importance of the role played by host genetics in disease progression within koalas remains unclear, with some studies suggesting that particular MHC alleles are associated with clinical disease outcomes [8, 18]. Given this, we assessed the level of variation of MHC class II alleles within our ocular diseased koalas and found that no two koalas had identical MHC class II alleles. However, we did identify the MHC class II DAb*21 allele in 6 (86%) of our koalas, which has previously been linked to chlamydial disease in wild koalas. We further identified DAb*19 allele in six (86%) of the ocular diseased koalas with five koalas having both the DAb*19 and DAb*21 allele. Surprisingly, we identified the MHC class II DBb*03 allele in four (57%) of our koalas but, interestingly, the absence of this allele has been associated with chlamydial disease in koalas [8]. In addition to the DBb alleles, DBb*05 allele was the most common allele, identified in five ocular diseased koalas. Due to the variation in MHC class II gene alleles identified within our diseased koalas, we were unable to determine if any one particular allele or set of alleles, was associated with the presence or severity of clinical ocular disease. However, given that MHC class II molecules influence antigen presentation to CD4^+^ T cells, which in turn influence B cell differentiation, antibody production and cytokine secretion, it is not surprising that we would then see differences in immune responses within each koala. This data provides further information on the identification of MHC class II alleles, identified within ocular diseased koalas, adding value to future research.

Additionally, as KoRV has been suggested to be linked with disease outcome for *Chlamydia* infected koalas [40, 42], we evaluated each koala’s KoRV-B status, comparing it to their ocular disease state. We identified KoRV-B in only 29% (2/7) of the ocular diseased koalas (K1 and K4) in this trial. Surprisingly, despite KoRV-B being associated with inducing immunosuppressive properties within the koala [88], this did not appear to have any effect on post-vaccine immune responses for koala K4, who showed some of the strongest humoral and cell mediated immune responses. However, koala K4 did exhibit low humoral and cell mediated responses pre-vaccination.

In conclusion, the vaccinated koalas from this trial showed a reduction in their clinical ocular disease state by six-weeks post-vaccination, without the intervention of antibiotic treatment. This has clearly demonstrated that a rMOMP vaccine can have a therapeutic effect on koala ocular disease. Vaccine development for preventing chlamydial infections in the koala is continuing, with this study now adding valuable insight into the therapeutic effects provided by a rMOMP vaccine on ocular disease. Given the detrimental effects resulting from the use of antibiotics in koalas, this study provides new evidence that a rMOMP vaccine could potentially be used in the treatment of ocular disease in the koala.

## Supporting information

S1 Fig Systemic IgG antibody response to rMOMP protein and whole *Chlamydia pecorum* elementary bodies. (TIF)

S2 Fig Mucosal IgA antibody response in left and right eye to rMOMP protein and whole *Chlamydia pecorum* elementary bodies. (TIF)

S3 Fig *Chlamydia pecorum* load. (TIF)

S1 Table Fold expression of IFN-γ, IL-6 and IL-17A compared to GAPDH in six koalas (K2, K3, K4, K5, K6 and K7) measured at pre-vaccination (week 0) and weekly for six-weeks post-vaccination. No sample = N/A; Below detection level = B/D.
(DOCX)

S2 Table Fold increase of IFN-γ, IL-6 and IL-17A in six koalas (K2, K3, K4, K5, K6 and K7) measured weekly from one to six-weeks post-vaccination. No sample = N/A; Below detection level = B/D.
(DOCX)
