# Supplementary figures and images for: Therapeutic effect of a Chlamydia pecorum recombinant major outer membrane protein vaccine on ocular disease in koalas (Phascolarctos cinereus)
Source: PLoS One. 2019 Jan 7;14(1):e0210245. doi: 10.1371/journal.pone.0210245 (PMC6322743; doi:10.1371/journal.pone.0210245)

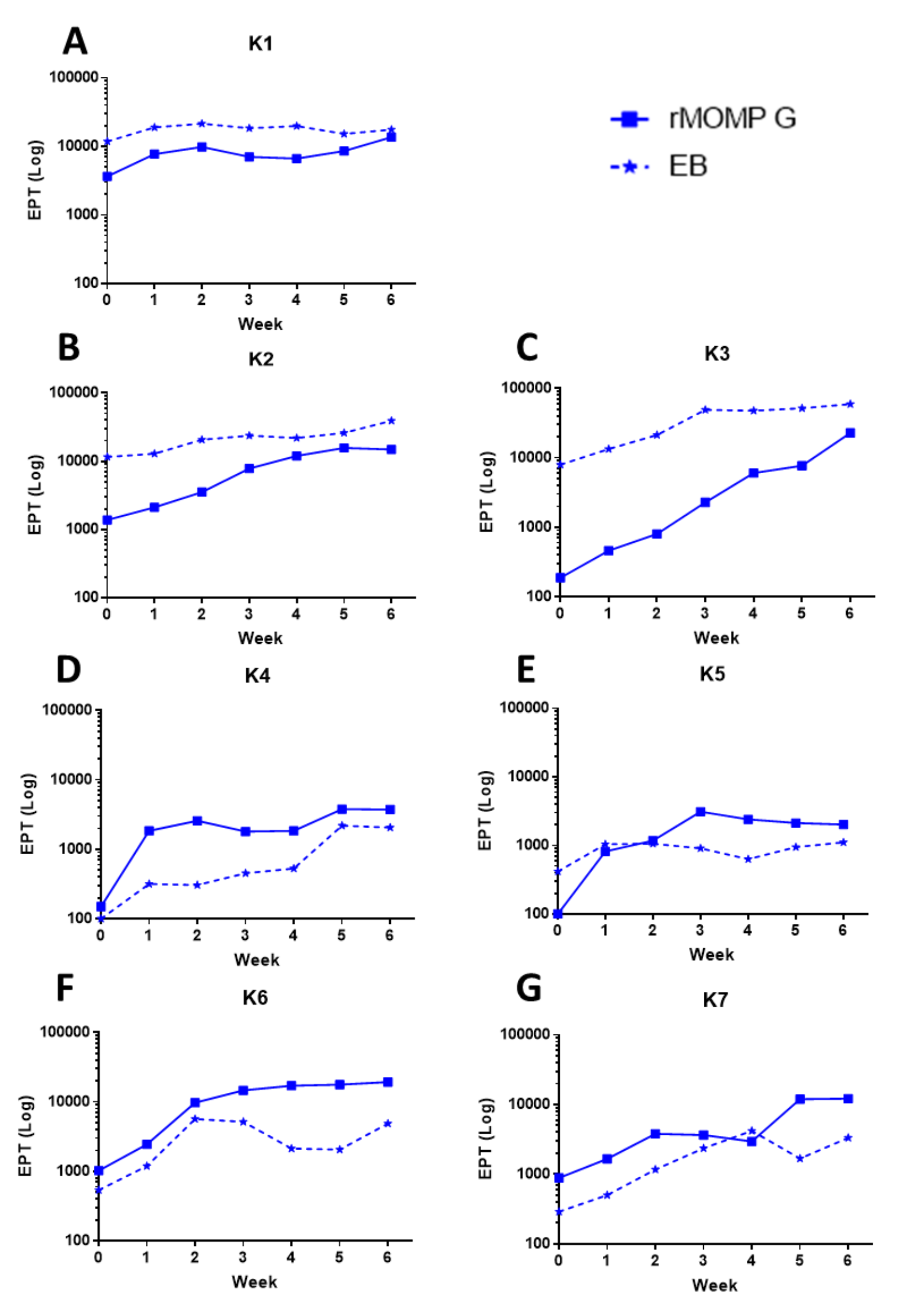

Supplement: S1 Fig — (TIF) [file pone.0210245.s001.tif]

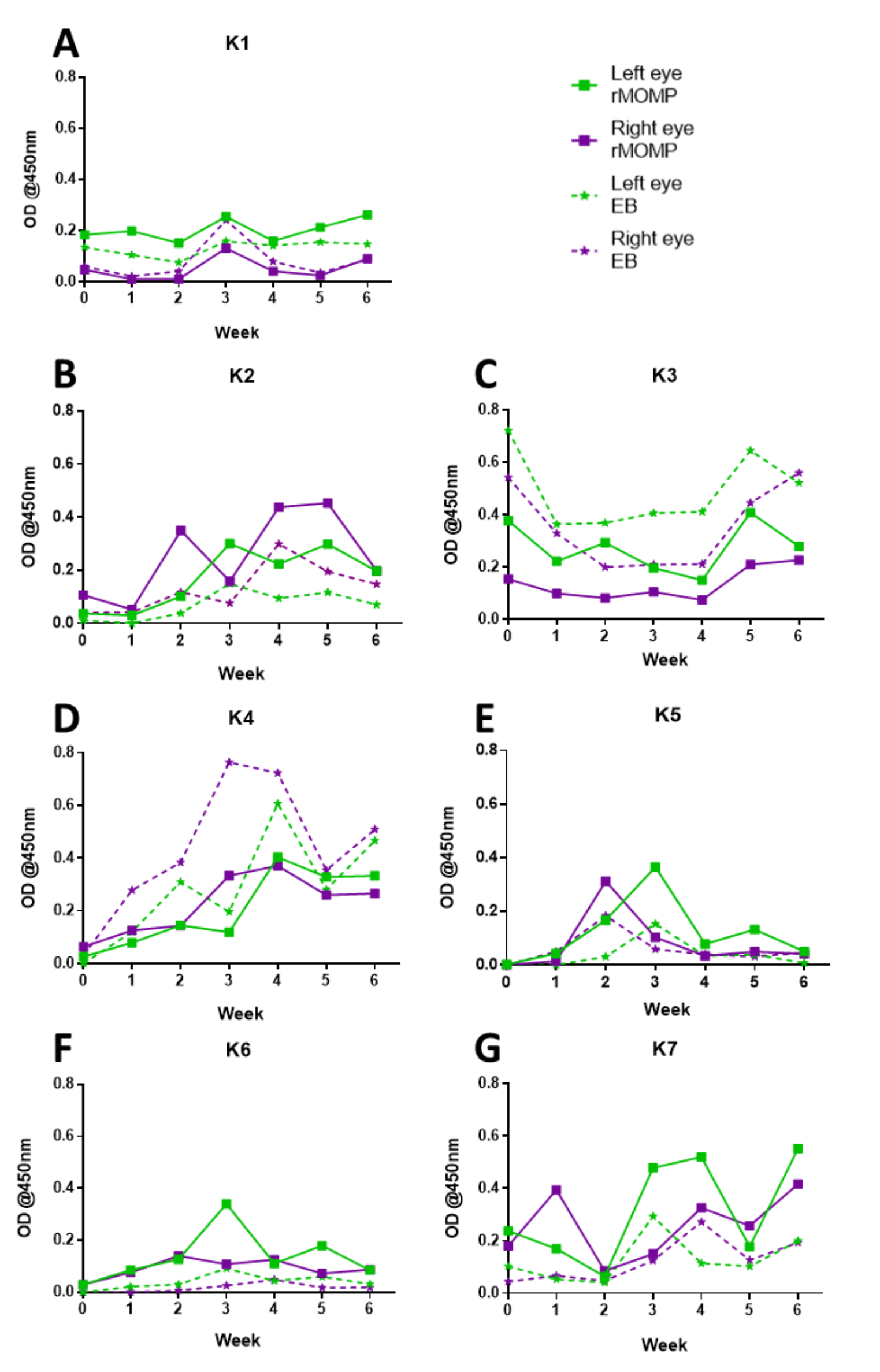

Supplement: S2 Fig — (TIF) [file pone.0210245.s002.tif]

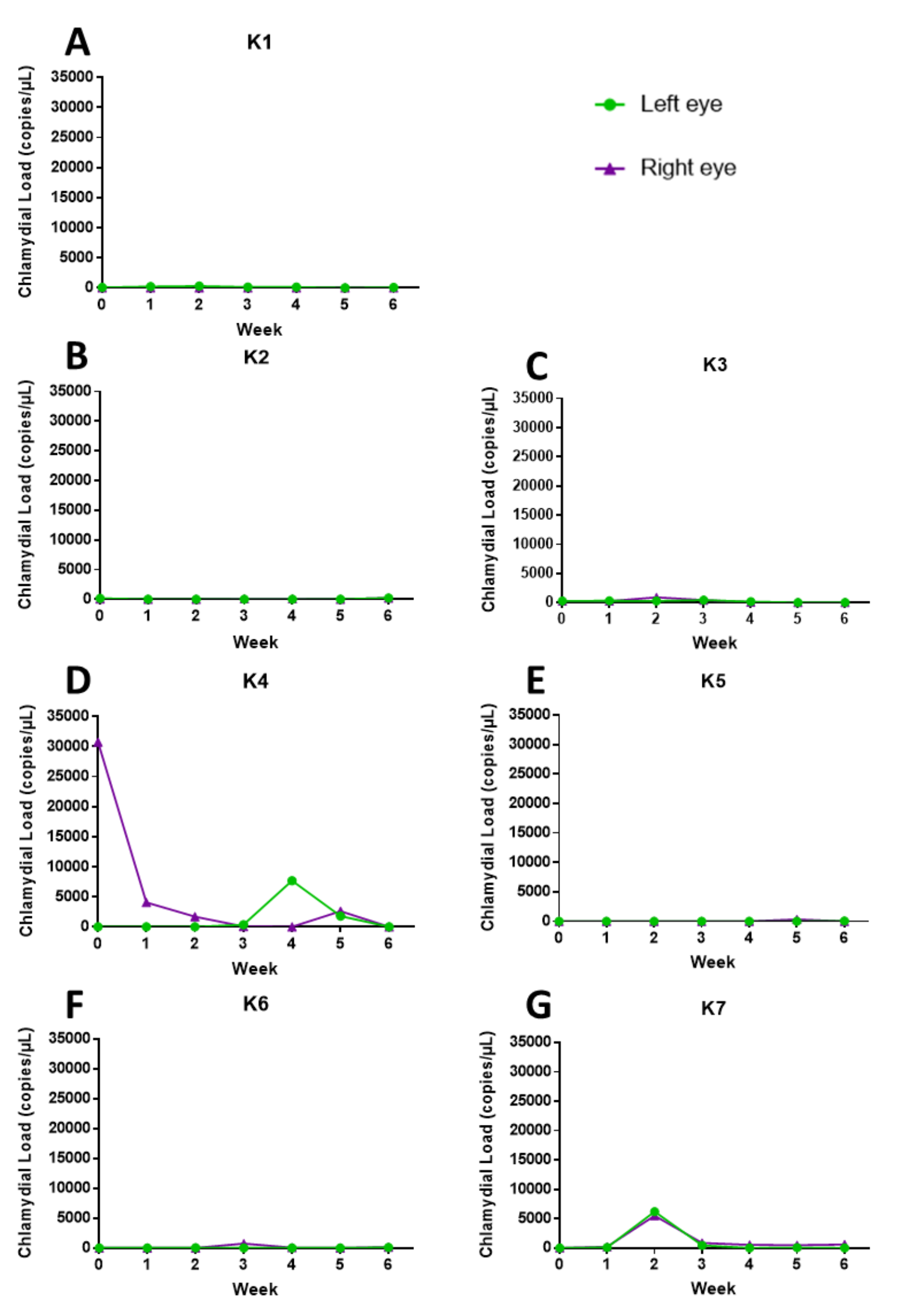

Supplement: S3 Fig — (TIF) [file pone.0210245.s003.tif]
