# Supplementary material for: Therapeutic effect of a Chlamydia pecorum recombinant major outer membrane protein vaccine on ocular disease in koalas (Phascolarctos cinereus)
Source: PLoS One. 2019 Jan 7;14(1):e0210245. doi: 10.1371/journal.pone.0210245 (PMC6322743; doi:10.1371/journal.pone.0210245)
Supplement: S1 Table — No sample = N/A; Below detection level = B/D. (DOCX) [file pone.0210245.s004.docx]

**S1 Table.** Fold expression of IFN-γ, IL-6 and IL-17A compared to GAPDH in six koalas (K2, K3, K4, K5, K6 and K7) measured at pre-vaccination (week 0) and weekly for six-weeks post-vaccination. No sample = N/A; Below detection level = B/D

**IFN-γ fold expression compared to GAPDH**

| **Koala** | **Week 0** | **Week 1** | **Week 2** | **Week 3** | **Week 4** | **Week 5** | **Week 6** |
| --- | --- | --- | --- | --- | --- | --- | --- |
| **K2** | 2.03 | 3.48 | 6.75 | 0.49 | B/D | 0.35 | 0.61 |
| **K3** | 0.98 | 11.43 | 0.58 | 0.01 | 4.96 | 0.35 | 1.10 |
| **K4** | 0.33 | 0.28 | 0.23 | 14.37 | 8.49 | 0.61 | 2.71 |
| **K5** | 0.14 | 0.07 | 0.31 | 0.03 | 0.02 | N/A | 0.22 |
| **K6** | 4.27 | 2.58 | 3.06 | 0.79 | 4.50 | 2.07 | N/A |
| **K7** | 0.39 | 2.39 | 0.39 | 0.09 | 0.10 | 1.16 | 0.39 |

**IL-6 fold expression compared to GAPDH**

| **Koala** | **Week 0** | **Week 1** | **Week 2** | **Week 3** | **Week 4** | **Week 5** | **Week 6** |
| --- | --- | --- | --- | --- | --- | --- | --- |
| **K2** | 1.14 | 1.55 | 4.30 | 0.81 | 0.90 | 1.19 | 0.71 |
| **K3** | 1.21 | 1.49 | 0.92 | 10.93 | 0.46 | 0.17 | 0.61 |
| **K4** | 0.01 | 1.61 | 0.45 | 4.03 | 1.35 | 0.22 | 2.33 |
| **K5** | 0.15 | 0.07 | 0.87 | 0.40 | 0.21 | N/A | 0.46 |
| **K6** | 1.07 | 1.04 | 1.23 | 0.60 | 1.57 | 1.59 | N/A |
| **K7** | 1.67 | 1.99 | 0.36 | 0.33 | 1.33 | 0.58 | 0.92 |

**IL-17A fold expression compared to GAPDH**

| **Koala** | **Week 0** | **Week 1** | **Week 2** | **Week 3** | **Week 4** | **Week 5** | **Week 6** |
| --- | --- | --- | --- | --- | --- | --- | --- |
| **K2** | 5.72 | 7.57 | B/D | 0.02 | 0.01 | 58.69 | 1.92 |
| **K3** | 2.90 | 11.88 | 17.88 | B/D | 42.37 | 1.47 | 7.62 |
| **K4** | 0.13 | 0.30 | 18.70 | 5.78 | 12.73 | 1.46 | 14.32 |
| **K5** | 4.68 | B/D | B/D | 0.08 | 0.42 | N/A | 0.11 |
| **K6** | 2.42 | 1.54 | 120.26 | 0.06 | 41.21 | 0.13 | N/A |
| **K7** | 20.75 | 7.39 | 2.21 | 2.12 | 0.02 | 2.98 | 4.69 |
