# Supplementary material for: Therapeutic effect of a Chlamydia pecorum recombinant major outer membrane protein vaccine on ocular disease in koalas (Phascolarctos cinereus)
Source: PLoS One. 2019 Jan 7;14(1):e0210245. doi: 10.1371/journal.pone.0210245 (PMC6322743; doi:10.1371/journal.pone.0210245)
Supplement: S2 Table — No sample = N/A; Below detection level = B/D. (DOCX) [file pone.0210245.s005.docx]

**S2 Table.** Fold increase of IFN-γ, IL-6 and IL-17A in six koalas (K2, K3, K4, K5, K6 and K7) measured weekly from one to six-weeks post-vaccination. No sample = N/A; Below detection level = B/D

**IFN-γ fold increase post-vaccination**

| **Koala** | **Week 1** | **Week 2** | **Week 3** | **Week 4** | **Week 5** | **Week 6** |
| --- | --- | --- | --- | --- | --- | --- |
| **K2** | 1.71 | 3.33 | 0.24 | B/D | 0.17 | 0.30 |
| **K3** | 11.66 | 0.59 | 0.01 | 5.06 | 0.36 | 1.12 |
| **K4** | 0.85 | 0.70 | 43.55 | 25.73 | 1.85 | 8.21 |
| **K5** | 0.50 | 2.21 | 0.21 | 0.14 | N/A | 1.57 |
| **K6** | 0.60 | 0.72 | 0.19 | 1.05 | 0.48 | N/A |
| **K7** | 6.13 | 1.00 | 0.23 | 0.26 | 2.97 | 1.00 |

**IL-6 fold increase post-vaccination**

| **Koala** | **Week 1** | **Week 2** | **Week 3** | **Week 4** | **Week 5** | **Week 6** |
| --- | --- | --- | --- | --- | --- | --- |
| **K2** | 1.36 | 3.77 | 0.71 | 0.79 | 1.04 | 0.62 |
| **K3** | 1.23 | 0.76 | 9.03 | 0.38 | 0.14 | 0.50 |
| **K4** | 161.00 | 45.00 | 403.00 | 135.00 | 22.00 | 233.00 |
| **K5** | 0.47 | 5.80 | 2.67 | 1.40 | N/A | 3.07 |
| **K6** | 0.97 | 1.15 | 0.56 | 1.47 | 1.49 | N/A |
| **K7** | 1.19 | 0.22 | 0.20 | 0.80 | 0.35 | 0.55 |

**IL-17A fold increase post-vaccination**

| **Koala** | **Week 1** | **Week 2** | **Week 3** | **Week 4** | **Week 5** | **Week 6** |
| --- | --- | --- | --- | --- | --- | --- |
| **K2** | 1.32 | B/D | 0.00 | 0.00 | 10.26 | 0.34 |
| **K3** | 4.10 | 6.16 | B/D | 14.61 | 0.51 | 2.63 |
| **K4** | 2.33 | 143.85 | 44.43 | 97.91 | 11.22 | 110.16 |
| **K5** | B/D | B/D | 0.02 | 0.09 | N/A | 0.02 |
| **K6** | 0.64 | 49.69 | 0.03 | 17.03 | 0.05 | N/A |
| **K7** | 0.36 | 0.11 | 0.10 | 0.00 | 0.14 | 0.23 |
